# Ideal and actual involvement of community pharmacists in health promotion and prevention: a cross-sectional study in Quebec, Canada

**DOI:** 10.1186/1471-2458-12-192

**Published:** 2012-03-15

**Authors:** Marie-Claude Laliberté, Sylvie Perreault, Nicole Damestoy, Lyne Lalonde

**Affiliations:** 1 Faculty of Pharmacy, Université de Montréal, C.P. 6128, Succ. Centre-ville, Montreal, Quebec H3C 3J7, Canada; 2 Équipe de recherche en soins de première ligne, Centre de santé et de services sociaux de Laval, 1755 René-Laennec Blvd, room D-S080, Laval, Quebec H7M 3L9, Canada; 3 Sanofi Aventis Endowment Chair in Drug Utilization, Faculty of Pharmacy, Université de Montréal, C.P. 6128, Succ. Centre-ville, Montreal, Quebec H3C 3J7, Canada; 4 Direction Prévention-Promotion, Centre de santé et de services sociaux de Laval, 800 Chomedey Blvd, Tour A, Laval, Quebec H7V 3Y4, Canada; 5 Direction de santé publique, Agence de la santé et des services sociaux de Laval, 800 Chomedey Blvd, Tour A, Laval, Quebec H7V 3Y4, Canada; 6 Faculty of Medicine, Université de Montréal, C.P. 6128, Succ. Centre-ville, Montreal, Quebec H3C 3J7, Canada; 7 Sanofi Aventis Endowment Chair in Ambulatory Pharmaceutical Care, Faculty of Pharmacy, Université de Montréal, C.P. 8128, Succ. Centre-ville, Montreal, Quebec H3C 3J7, Canada

**Keywords:** Community pharmacists, Cross-sectional study, Health promotion, Prevention, Public health

## Abstract

**Background:**

An increased interest is observed in broadening community pharmacists' role in public health. To date, little information has been gathered in Canada on community pharmacists' perceptions of their role in health promotion and prevention; however, such data are essential to the development of public-health programs in community pharmacy. A cross-sectional study was therefore conducted to explore the perceptions of community pharmacists in urban and semi-urban areas regarding their ideal and actual levels of involvement in providing health-promotion and prevention services and the barriers to such involvement.

**Methods:**

Using a five-step modified Dillman's tailored design method, a questionnaire with 28 multiple-choice or open-ended questions (11 pages plus a cover letter) was mailed to a random sample of 1,250 pharmacists out of 1,887 community pharmacists practicing in Montreal (Quebec, Canada) and surrounding areas. It included questions on pharmacists' ideal level of involvement in providing health-promotion and preventive services; which services were actually offered in their pharmacy, the employees involved, the frequency, and duration of the services; the barriers to the provision of these services in community pharmacy; their opinion regarding the most appropriate health professionals to provide them; and the characteristics of pharmacists, pharmacies and their clientele.

**Results:**

In all, 571 out of 1,234 (46.3%) eligible community pharmacists completed and returned the questionnaire. Most believed they should be very involved in health promotion and prevention, particularly in smoking cessation (84.3%); screening for hypertension (81.8%), diabetes (76.0%) and dyslipidemia (56.9%); and sexual health (61.7% to 89.1%); however, fewer respondents reported actually being very involved in providing such services (5.7% [lifestyle, including smoking cessation], 44.5%, 34.8%, 6.5% and 19.3%, respectively). The main barriers to the provision of these services in current practice were lack of: time (86.1%), coordination with other health care professionals (61.1%), staff or resources (57.2%), financial compensation (50.8%), and clinical tools (45.5%).

**Conclusions:**

Although community pharmacists think they should play a significant role in health promotion and prevention, they recognize a wide gap between their ideal and actual levels of involvement. The efficient integration of primary-care pharmacists and pharmacies into public health cannot be envisioned without addressing important organizational barriers.

## Background

Recently, there has been an increased interest in broadening community pharmacists' functions toward playing a greater role in public health [1]. Community pharmacies are often considered an ideal site for credible counseling for a large segment of the population because pharmacists are accessible, have frequent contact with the public, have extended opening hours, and are widely distributed geographically [1-3]. Given the increasing stresses on the health care system due to an aging population and the consequent rise in the prevalence and incidence of chronic diseases, the shift toward a wider public-health role for pharmacists should be accentuated.

Health-promotion and preventive services refer to public health services, which relate to the improvement of the general health of the population through interventions aiming at promoting health and wellbeing (e.g. nutrition, physical activity), preventing diseases (e.g. smoking cessation, immunization, travel health), identifying ill individuals (e.g. screening and case finding) and maintaining health of those with chronic conditions (e.g. diabetes, hypertension) [4,5]. Public health interventions act on factors influencing the health of the population as a whole or subgroups of this population rather than separate individuals, and generally take place before the onset of health problems [4]. Throughout this paper, clinical services such as medication reviews were not considered as health-promotion or preventive services since they target specific individuals.

Studies from many countries have demonstrated the benefits of pharmacy services on a wide range of important public-health issues [5]: notably in smoking cessation [6,7], diabetes [8,9], hypertension [9,10], dyslipidemia [9], contraception [11], osteoporosis [12-14], and immunization [15,16]. A recent Cochrane review confirmed the importance of the pharmacist's role in therapeutic management and patient counseling [17]. The broader involvement of community pharmacists is thus deemed a valuable option in addressing several public-health issues. In some countries, such as the United Kingdom, pharmacists are integrated into public-health programs [18]. In contrast, Quebec's public-health program does not stress the role of pharmacists as primary-care providers in this area [4].

To date, little information has been gathered in Canada on community pharmacists' perceptions of their role in health promotion and prevention. However, such data are essential to the development of public-health programs in community pharmacy. In this study, we documented the perceptions of community pharmacists in urban and semi-urban areas regarding their ideal and actual levels of involvement in the provision of health-promotion and preventive services, as well as the barriers that limit their involvement.

## Results

Of the 1,250 pharmacists selected, eight did not work in community pharmacy. Twelve questionnaires were returned undelivered because the address was wrong. Following the first questionnaire mailing, we were informed that one pharmacist had retired, one was on maternity leave, and one declined (verbally) to take part. Ultimately, 577 completed questionnaires were returned. However, six were returned by non-eligible pharmacists (four did not work in community pharmacy, and two were not working in a traditional community pharmacy), for a total of 571 questionnaires returned out of 1,234 eligible pharmacists (response rate: 46.3%). More specifically, 249 questionnaires (43.6%) were received after the first questionnaire mailing, 114 (20.0%) after the postcard mailing, 149 (26.1%) after the second questionnaire mailing, and 59 (10.3%) after the third questionnaire mailing. The response rates for individual questions ranged from 87% to 100%, and 99% of questions had a response rate over 90%.

As reported in Table 1, the respondents were in majority women (63.2%), were staff pharmacists (65.3%), and reported having completed a mean 31 hours of continuing education during the past year. Their pharmacies were associated with a chain or a corporate banner (80.4%) and/or adjacent to a medical clinic (28.7%). The most prevalent clienteles were elderly patients and families of average to high socio-economic status. Furthermore, 53.5% of pharmacists reported that a nurse was present in the pharmacy for a mean 47 hours per month. Nutritionists (69 pharmacists) and naturopaths (11 pharmacists) were among other common health professionals employed in the pharmacy. Based on information from the *Ordre des pharmaciens du Québec *(OPQ), our respondents were similar to community pharmacists working in Quebec in terms of sex (62.4% women in OPQ) and pharmacist status (63.9% staff pharmacists in OPQ).

**Table 1:** Characteristics of pharmacists and pharmacies

Characteristics	Total (n = 571)
**Characteristics of pharmacists**	
Sex, n (%)	
Men	210 (36.8)
Women	361 (63.2)
Practice region, n (%)	
Montreal	184 (32.2)
Laval	51 (8.9)
Laurentides	75 (13.1)
Lanaudière	59 (10.3)
Montérégie	149 (26.1)
Estrie	30 (5.3)
Outaouais	23 (4.0)
Years since graduation, n (%)	
≤ 10 years	230 (40.7)
11-20 years	135 (23.9)
21-30 years	106 (18.8)
≥ 31 years	94 (16.6)
Pharmacist status, n (%)	
Owner pharmacist--single owner	78 (13.7)
Owner pharmacist--owner partner	82 (14.4)
Staff pharmacist	371 (65.3)
Replacement pharmacist	27 (4.8)
Staff and relief pharmacist	10 (1.8)
Hours worked per week, mean (SD^a^)	33.6 (9.9)
Hours of continuing education during past year, mean (SD)	31.2 (37.3)
Private consultations in pharmacy privacy area per week, mean (SD)	22.0 (47.5)
**Characteristics of pharmacies**	
Type of pharmacy, n (%)^b^	
Pharmacy adjacent to a medical clinic	163 (28.7)
Pharmacy associated with a chain or corporate banner	456 (80.4)
Independent pharmacy	16 (2.8)
Pharmacy associated with a supermarket or superstore	39 (6.9)
Pharmacy in a retirement home	21 (3.7)
Prescriptions filled per day, n (%)	
< 250 prescriptions	147 (25.9)
250 to 500 prescriptions	197 (34.7)
> 500 prescriptions	224 (39.4)
Characteristics of most prevalent patients at the pharmacy, n(%)^b^	
Adults (18-49 years)	44 (8.1)
Older adults (50-65 years)	33 (6.1)
Elderly patients (> 65 years)	347 (63.9)
Young families	73 (13.4)
Families	159 (29.3)
All ages (various)	57 (10.5)
Other^c^	86 (15.8)
Low to average socio-economic status	143 (26.3)
Average to high socio-economic status	273 (50.3)
High socio-economic status	43 (7.9)
Availability of other health professionals in the pharmacy, n(%)^b^	
Nurse	289 (53.5)
Nutritionist	69 (12.8)
Other^d^	27 (5.0)
None	229 (42.4)
Hours per month of availability of other health professionals	
in the pharmacy, mean (SD)	
Nurse	47.4 (45.1)
Nutritionist	9.6 (6.5)

^a^*SD *standard deviation

^b^More than one item could be checked.

^c^Other includes multicultural background (n = 23); workers/professionals (n = 13); children (n = 11); students (n = 9); patients with mental health problems (n = 8); rural background (n = 5); drug abuse (n = 5); patients from walk-in clinics or emergency rooms (n = 5); patients in long-term care facilities or nursing homes (n = 4); tourists (n = 1); patients with organ transplant or dialysis (n = 1); and homosexuals (n = 1).

^d^Other includes naturopath (n = 11); breast-feeding counselor (n = 5); psychotherapist (n = 2); physiotherapist (n = 3); kinesiologist (n = 1); occupational therapist (n = 1); massage therapist (n = 1); homeopath (n = 1); respiratory therapist (n = 1); and medical laboratory technologist (n = 1).

As Table 2 shows, most respondents believed they should be either "very involved" or "involved" in providing all health-promotion and preventive services. The majority reported they should ideally be "very involved" in smoking cessation (84.3%); screening for hypertension (81.8%), diabetes (76.0%) and dyslipidemia (56.9%); and sexual health (61.7% to 89.1%). Most considered they should be "involved" in providing information and counseling about physical activity (71.1%), healthy eating (68.8%), weight management (63.4%), and alcohol consumption (63.8%). In contrast, 54.3% and 28.5% of pharmacists, respectively, considered they should have "little involvement" or "no involvement at all" in counseling on dental health and screening for suicide risk. The proportion of respondents who reported their community pharmacy as being actually "very involved" in each service was 5.7% for lifestyle-related activities, 44.5% for screening for hypertension, 34.8% for screening for diabetes, 6.5% for screening for dyslipidemia and 19.3% for sexual health. Most respondents reported their pharmacy as being either "involved" or "little involved" in lifestyle-related activities (84.5%), screening for dyslipidemia (57.8%), sexual health (74.5%) and infectious diseases and immunization (72.4%). Most appropriate primary-care providers for preventive counseling or screening were primary-care physicians, community pharmacists and nurses. They also deemed kinesiologists, nutritionists and physiotherapists well placed to offer lifestyle-related services.

**Table 2:** Ideal and actual levels of involvement of community pharmacists and most appropriate primary-care providers for these services

Services	Very involved n (%)	Involved n (%)	Little involved n (%)	Not at all involved n (%)
**Ideal level of involvement**				
**Lifestyle**				
Smoking cessation	478 (84.3)	85 (15.0)	4 (0.7)	0 (0)
Physical-activity promotion	94 (16.6)	403 (71.1)	68 (12.0)	2 (0.4)
Healthy eating	97 (17.2)	389 (68.8)	76 (13.5)	3 (0.5)
Weight management	128 (22.6)	359 (63.4)	75 (13.3)	4 (0.7)
Alcohol consumption	80 (14.1)	361 (63.8)	117 (20.7)	8 (1.4)
Dental health	27 (4.8)	231 (40.9)	281 (49.7)	26 (4.6)
**Screening for:**				
Hypertension	464 (81.8)	96 (16.9)	7 (1.2)	0 (0)
Diabetes	431 (76.0)	127 (22.4)	9 (1.6)	0 (0)
Dyslipidemia	322 (56.9)	217 (38.3)	24 (4.2)	3 (0.5)
Risk of suicide	138 (24.4)	266 (47.1)	148 (26.2)	13 (2.3)
**Sexual health**				
Emergency oral contraception	505 (89.1)	58 (10.2)	3 (0.5)	1 (0.2)
Contraception	361 (63.7)	191 (33.7)	15 (2.6)	0 (0)
Counseling with partners when initiating treatment for sexually transmitted diseases	350 (61.7)	194 (34.2)	21 (3.7)	2 (0.4)
**Infectious diseases and immunization**				
Travel health	242 (42.8)	276 (48.8)	44 (7.8)	4 (0.7)
Needle-exchange programs	259 (45.8)	236 (41.8)	60 (10.6)	10 (1.8)
Immunization programs	163 (28.8)	313 (55.4)	82 (14.5)	7 (1.2)
**Actual level of involvement**				
Lifestyle	32 (5.7)	232 (41.3)	243 (43.2)	55 (9.8)
Screening for hypertension	252 (44.5)	267 (47.2)	42 (7.4)	5 (0.9)
Screening for diabetes	198 (34.7)	255 (44.7)	100 (17.5)	17 (3.0)
Screening for dyslipidemia	37 (6.5)	150 (26.5)	177 (31.3)	202 (35.7)
Sexual health	109 (19.3)	274 (48.5)	147 (26.0)	35 (6.2)
Infectious diseases and immunization	48 (8.6)	189 (33.8)	216 (38.6)	107 (19.1)

**Most appropriate providersa**	**Primary care physicians n (%)**	**Community pharmacists n (%)**	**Nurses n (%)**	**Other or nonen** **n (%)**

Lifestyle	327 (61.0)	379 (70.7)	354 (66.0)	166 (31.0)b
Screening for hypertension	399 (71.6)	520 (93.4)	429 (77.0)	22 (4.0)c
Screening for dyslipidemia	443 (83.1)	328 (61.5)	311 (58.3)	22(4.2)d
Screening for diabetes	419 (75.2)	494 (88.7)	442 (79.4)	27 (4.9)e
Sexual health	427 (77.6)	488 (88.7)	416 (75.6)	13 (2.3)f
Infectious diseases and immunization	371 (70.3)	313 (59.3)	450 (85.2)	17 (3.2)g

^a^More than one item could be checked.

^b^Other includes kinesiologists (n = 48); nutritionists (n = 46); physiotherapists (n = 28); personal trainers in gyms (n = 12); dieticians (n = 12); occupational therapists (n = 11); physical educators in schools (n = 10); all health professionals as multidisciplinary teams (n = 7); government (n = 5); public-health agencies (n = 4); technical assistants (n = 2); local community service centre (CLSC) (n = 2); community support groups (n = 2); technicians (n = 2); recreation consultants (n = 1); and pharmacy students (n = 1).

^c^Other includes technical assistants (n = 9); pharmacy students (n = 3); technicians (n = 3); the patient him/herself (n = 3); and CLSC (n = 2).

^d^Other includes nutritionists (n = 6); technical assistants (n = 1); CLSC (n = 1); dieticians (n = 1); and pharmacy students (n = 1).

^e^Other includes technical assistants (n = 8); nutritionists (n = 7); dieticians (n = 3); diabetes clinic (n = 3); CLSC (n = 2); physiotherapists (n = 1); technicians (n = 1); and the patient him/herself (n = 1).

^f^Other includes CLSC (n = 5); schools (n = 3); public-health agencies (n = 1); and sexologists (n = 1).

^g^Other includes travel clinic (n = 5); CLSC (n = 4); and public-health agencies (n = 1).

As Table 3 shows, the majority of respondents identified the pharmacist as the main provider of health-promotion and preventive services in their pharmacy, though nurses and technical assistants were also frequently cited. Most pharmacists reported that preventive services are given a "few times per week" or a "few times per month" regarding: lifestyle (69.5%), screening for hypertension (96.6%), screening for diabetes (86.2%), and counseling on sexual health (81.5%). Activities related to infectious diseases and immunization were reported for the most part to take place a "few times per month" or a "few times per year" (61.8%); 36.8% of respondents indicated that screening for dyslipidemia never occurred. Consultations were reported to last 10 minutes or less by most pharmacists. When cases are detected during screening, most pharmacists said the report is given to the patient only (67.4% for hypertension, 35.7% for dyslipidemia and 67.9% for diabetes); smaller percentages said the report is given to both the patient and the primary-care physician (33.5% for hypertension, 18.6% for dyslipidemia and 31.4% for diabetes).

**Table 3:** Characteristics of health-promotion and preventive services

	Lifestyle	Screening for hypertension	Screening for dyslipidemia	Screening for diabetes	Sexual health	Infectious diseases and immunization
**Main person(s) providing health-promotion and preventive services, n (%)^a^**						
Pharmacist	432 (80.0)	497 (88.0)	202 (38.5)	462 (82.2)	522 (94.2)	348 (65.7)
Technical assistant	32 (5.9)	177 (31.3)	17 (3.2)	151 (26.9)	13 (2.3)	19 (3.6)
Nurse	143 (26.5)	215 (38.1)	197 (37.6)	241 (42.9)	58 (10.5)	9 (1.7)
Other	23 (4.3)^b^	21 (3.7)^c^	9 (1.7)^d^	11 (2.0)^e^	3 (0.5)^f^	2 (0.4)^g^
None	73 (13.5)	5 (0.9)	168 (32.1)	11 (2.0)	28 (5.1)	80 (15.1)
**Frequency with which health-promotion and preventive services are provided, n (%)^a^**						
Few times per week	203 (38.2)	465 (82.6)	96 (18.4)	301 (53.8)	249 (45.4)	116 (22.0)
Few times per month	166 (31.3)	79 (14.0)	133 (25.5)	181 (32.4)	198 (36.1)	171 (32.4)
Few times per year	94 (17.7)	21 (3.7)	107 (20.5)	77 (13.8)	63 (11.5)	155 (29.4)
Never	74 (13.9)	6 (1.1)	192 (36.8)	9 (1.6)	40 (7.3)	89 (16.9)
**Duration of consultations related to health promotion and prevention, n (%)^a^**						
Less than 5 minutes	255 (48.5)	157 (27.9)	71 (13.8)	97 (17.3)	128 (23.4)	149 (28.6)
5-10 minutes	153 (29.1)	324 (57.7)	151 (29.3)	301 (53.8)	279 (50.9)	180 (34.5)
11-15 minutes	51 (9.7)	80 (14.2)	85 (16.5)	139 (24.8)	99 (18.1)	85 (16.3)
More than 15 minutes	19 (3.6)	10 (1.8)	25 (4.9)	39 (7.0)	15 (2.7)	29 (5.6)
No consultation provided	72 (13.7)	7 (1.2)	188 (36.5)	12 (2.1)	32 (5.8)	87 (16.7)
**Type(s) of follow-up for cases detected during screening, n (%)^a^**						
Report given to patient only	0 (0)	372 (67.4)	178 (35.7)	374 (67.9)	0 (0)	0 (0)
Report given to primary-care physician	0 (0)	25 (4.5)	12 (2.4)	22 (4.0)	0 (0)	0 (0)
only	0 (0)	185 (33.5)	93 (18.6)	173 (31.4)	0 (0)	0 (0)
Report given to patient and primary-care physician	0 (0)	4 (0.7)	2 (0.4)	6 (1.1)	0 (0)	0 (0)
Link with the *Centre de santé et de services sociaux *Not applicable	0 (0)	28 (5.1)	232 (46.5)	36 (6.5)	0 (0)	0 (0)

^a^More than one item could be checked.

^b^Other includes nutritionists (n = 16); pharmacy students (n = 5); and dieticians (n = 2).

^c^Other includes blood-pressure device available at the pharmacy (n = 9); pharmacy students (n = 8); nursing students (n = 3); and over-the-counter medications salesperson (n = 1).

^d^Other includes pharmacy students (n = 6); nutritionist (n = 2); and dietician (n = 1).

^e^Other includes pharmacy students (n = 4); nutritionist (n = 3); over-the-counter medications salesperson (n = 2); nursing students (n = 1); and the patient in the presence of the pharmacist (n = 1).

^f^Other includes pharmacy students (n = 3).

^g^Other includes pharmacy students (n = 1) and travel clinic at the pharmacy (n = 1).

Pharmacists were also asked to report the actual specific activities provided in their pharmacy. As indicated in Table 4, the majority reported distributing written information, providing personalized counseling when dispensing medications and referring patients to external resources. A large proportion of respondents said personalized follow-up for smoking cessation (44.5%), hypertension (53.7%), diabetes (45.0%), and emergency oral contraception (40.7%) were provided. Many pharmacists provided no prevention activities regarding dental health (29.3%), suicide risk (27.6%) or needle exchange (35.2%). Many tasks were performed by a nurse or a nutritionist; 31 pharmacists thus reported that immunization was conducted by a nurse. Several pharmacists reported having a collective prescription for smoking cessation (39 pharmacists). Collective prescriptions enable authorized professionals, usually pharmacists or nurses, to perform certain tasks (for example, requesting laboratory tests and adjusting medication dosage) without first having to obtain an individual prescription from a physician [19].

**Table 4:** Specific activities conducted in health-promotion and preventive services in community pharmacies^a^

Services	Distribution of written information n (%)	Personalized counseling when Dispensing medications n (%)	Screening^b^ n (%)	Referral to external resources n (%)	Personalized follow-up or private consultation n (%)	Other n (%)	None n (%)
**Lifestyle**							
Smoking cessation	386 (68.2)	509 (89.9)	193 (34.1)	283 (50.0)	252 (44.5)	44 (7.8)^c^	0 (0)
Physical-activity promotion	154 (27.8)	349 (63.0)	20 (3.6)	169 (30.5)	23 (4.2)	9 (1.6)^d^	51 (9.2)
Healthy eating	304 (54.0)	355 (63.1)	43 (7.6)	297 (52.8)	30 (5.3)	29 (5.2)^e^	16 (2.8)
Weight management	150 (27.3)	277 (50.4)	42 (7.6)	257 (46.7)	23 (4.2)	25 (4.5)^f^	72 (13.1)
Alcohol consumption	75 (13.6)	309 (56.1)	32 (5.8)	155 (28.1)	16 (2.9)	3 (0.5)^g^	116 (21.1)
Dental health	47 (8.7)	180 (33.1)	13 (2.4)	262 (48.3)	7 (1.3)	4 (0.7)^h^	159 (29.3)
**Screening for:**							
Hypertension	344 (60.8)	480 (84.8)	417 (73.7)	186 (32.9)	304 (53.7)	35 (6.2)^i^	6 (1.1)
Diabetes	364 (64.5)	489 (86.7)	351 (62.2)	246 (43.6)	254 (45.0)	33 (5.9)^j^	8 (1.4)
Dyslipidemia	319 (57.1)	471 (84.3)	140 (25.0)	161 (28.8)	132 (23.6)	22 (3.9)^k^	21 (3.8)
Risk of suicide	81 (15.1)	190 (35.4)	44 (8.2)	239 (44.6)	67 (12.5)	2 (0.4)^l^	148 (27.6)
**Sexual health**							
Emergency oral contraception	227 (40.2)	497 (88.0)	122 (21.6)	137 (24.2)	230 (40.7)	26 (4.6)^m^	10 (1.8)
Contraception	214 (38.1)	510 (90.7)	58 (10.3)	220 (39.1)	105 (18.7)	2 (0.4)^n^	11 (2.0)
Counseling with partners when initiating treatment for sexually transmitted diseases	111 (19.8)	488 (87.0)	36 (6.4)	166 (29.6)	51 (9.1)	0 (0)	30 (5.3)
**Infectious diseases and immunization**							
Travel health	241 (43.0)	412 (73.4)	78 (13.9)	319 (56.9)	58 (10.3)	40 (7.1)^o^	28 (5.0)
Needle exchange programs	91 (16.9)	163 (30.4)	34 (6.3)	117 (21.8)	14 (2.6)	67 (12.5)^p^	189 (35.2)
Immunization programs	207 (37.3)	267 (48.1)	50 (9.0)	274 (49.4)	45 (8.1)	45 (8.1)^q^	82 (14.8)

^a^More than one item could be checked.

^b^Screening may be performed using a specific measure (e.g. blood-pressure measurement) and/or by documenting other risk factors (e.g. medication review, interview with the patient).

^c^Other includes collective prescription (n = 39); health clinic at the pharmacy (n = 1); service offered by a nurse at the pharmacy (n = 1); oral presentations (n = 1); referral to the "iQuitnow" organization (n = 1); and answers to patients' questions (n = 1).

^d^Other includes answers to patients' questions (n = 5); service offered by a nurse at the pharmacy (n = 2); oral presentations (n = 1); and referral to a website (n = 1).

^e^Other includes service offered by a nutritionist at the pharmacy (n = 13); counseling by a nurse at the pharmacy (n = 7); answers to patients' questions (n = 4); health clinic at the pharmacy (n = 3); referral to a website (n = 1); and distribution of Canada's Food Guide (n = 1).

^f^Other includes service by a nurse at the pharmacy (n = 10); service by a nutritionist at the pharmacy (n = 7); answers to patients' questions (n = 3); distribution of Canada's Food Guide (n = 1); referral to a website (n = 1); body-mass index measurement (n = 1); counseling on over-the-counter medications (n = 1); and health clinic at the pharmacy (n = 1).

^g^Other includes answers to patients' questions (n = 2) and referral to a website (n = 1).

^h^Other includes counseling on over-the-counter products (n = 2) and answers to patients' questions (n = 2).

^i^Other includes service by a nurse at the pharmacy (n = 16); blood-pressure measurement at the pharmacy (n = 8); ambulatory blood pressure monitoring (n = 6); health clinic at the pharmacy (n = 3); "Tension Attention" program (n = 1); and follow-up with the physician (n = 1).

^j^Other includes service by a nurse at the pharmacy (n = 19); health clinic at the pharmacy (n = 5); glycemia measurement at the pharmacy (n = 3); oral presentations (n = 2); service by a nutritionist at the pharmacy (n = 2); collective prescription for insulin monitoring (n = 1); and follow-up with the physician (n = 1).

^k^Other includes service by a nurse at the pharmacy (n = 16); Cholestech device (n = 2); health clinic at the pharmacy (n = 1); oral presentations (n = 1); service by a nutritionist at the pharmacy (n = 1); and collective prescription (n = 1).

^l^Other includes control of medication distribution and quantities (n = 1) and counseling by a nurse at the pharmacy (n = 1).

^m^Other includes prescription of emergency oral contraception (n = 26).

^n^Other includes counseling when prescription refills are late (n = 1) and suggestion of pregnancy test (n = 1).

^o^Other includes service by a nurse at the pharmacy (n = 20); collective prescriptions (n = 8); travel clinic near the pharmacy (n = 4); counseling on over-the-counter medications (n = 3); vaccination/health clinic at the pharmacy (n = 2); referral to a website (n = 2); and oral presentation (n = 1).

^p^Other includes needle-exchange program or distribution of clean needles (n = 27); distribution of toolkits (n = 17); needle recovery (n = 10); distribution of containers (n = 7); sale of needles (n = 3); poster in the pharmacy (n = 1); methadone program (n = 1); and collaborative service with the CLSC (n = 1).

^q^Other includes vaccination by a nurse at the pharmacy (n = 31) and influenza vaccination at the pharmacy (n = 14).

**Figure 1:**
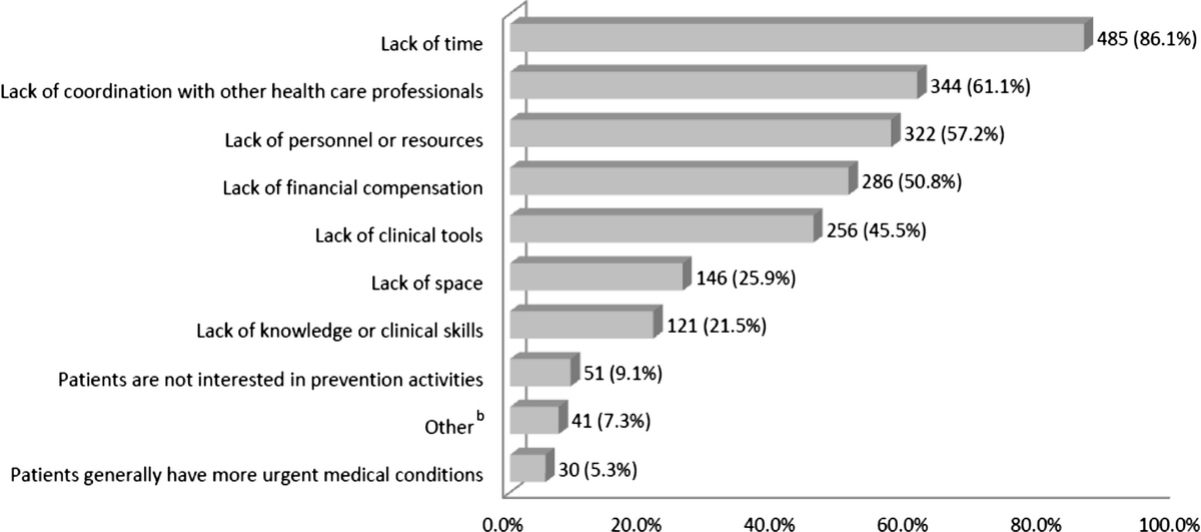
**Self-identified barriers to the provision of health-promotion and preventive services in current pharmacy practice^a^**.

^a^More than one item could be checked.

^b^Other includes limited prescription rights for pharmacists and lack of collective prescriptions (n = 6); patients are often in a rush and don't have time for prevention activities (n = 3); patients are not "open" to change (n = 3); too many technical tasks performed by the pharmacist (n = 2); patients do not think of pharmacists for prevention activities (n = 2); no standardized practice model (n = 2); lack of access to lab-test results and other patient information (n = 2); pharmacists are overworked (n = 2); nurses already perform some of these activities (n = 2); lack of a closed office at the pharmacy (n = 1); low uptake of pharmacist's suggestions by physicians (n = 1); home visits are expensive (n = 1); pharmacy owners not often present at the pharmacy (n = 1); pharmacist shortage (n = 1); patients don't like to be "criticized" about their lifestyle (n = 1); relief pharmacists don't know the pharmacy's patients very well (n = 1); the logistics of implementation in general (n = 1); most of the pharmacy's patients are not regular patients (n = 1); lack of patient knowledge about the benefits of prevention (n = 1); lack of tools for patients (n = 1); pharmacy's patients do not need preventive activities because they are already educated about the subject (n = 1); lack of external resources to which patients can be referred (n = 1); lack of budget for prevention activities at the pharmacy (n = 1); lack of competent and stable staff (n = 1); lack of interest by pharmacy owners (n = 1); and the topic of prevention is very broad, so pharmacists provide counseling on a little bit of everything but about nothing in depth (n = 1).

As reported in Figure 1 the main barriers to providing health-promotion and preventive services in their current practice were lack of time (86.1%), lack of coordination with other health care professionals (61.1%), lack of staff or resources (57.2%), lack of financial compensation (50.8%), and lack of clinical tools (45.5%). Six pharmacists also indicated that their limited prescription rights and the lack of collective prescriptions further hampered their involvement in prevention.

## Discussion

Community pharmacists in Montreal (Quebec, Canada) and surrounding areas perceive their potential role in health promotion and prevention as very significant, particularly in smoking cessation, screening for hypertension, diabetes and dyslipidemia, and counseling on sexual health. However, there is a wide gap between their ideal and actual levels of involvement. Most pharmacists believe they should be very involved in screening for hypertension (81.8%), diabetes (76.0%) and dyslipidemia (56.9%); in fact, though, only a minority is very involved (44.5%, 34.8% and 6.5%, respectively). Health-promotion and preventive services in pharmacy are provided largely by pharmacists and, to a lesser extent, by nurses and technical assistants. In fact, more than half of surveyed pharmacists reported that a nurse was present in their pharmacy. The services consist mainly of distributing written information, providing counseling when dispensing medications and referring patients to external resources. When offered, such services are provided relatively often and usually take 10 minutes or less. There are several barriers that limit pharmacists' involvement in health promotion and prevention, including lack of time, lack of coordination with other health care professionals, lack of staff or resources, lack of financial compensation, and lack of clinical tools.

Similarly to the present study, the results of a cross-sectional mail survey of community pharmacists in British Columbia published in 1994 showed that pharmacists are mostly involved in activities directly related to the dispensing or selling of medications and have less intense involvement in health education and disease prevention [2]. A 1996 cross-sectional mail survey of community pharmacists practicing in the province of Quebec found that, although only few pharmacists reported routinely performing prevention activities, over 90% believed that integrating prevention into their practices was important [1]. Similarly, a Web-based survey of pharmacists across Canada reported that, although pharmacists currently spend most of their time on dispensing duties, over 60% believed it was time to assume new responsibilities, and more than 70% wanted to expand their roles in various fields including public health outreach (e.g. working with communities and patients to focus on health promotion, disease prevention and chronic disease management) within five years [20]. Finally, a recent systematic review on the beliefs and attitudes of pharmacists regarding pharmaceutical public health showed that, although most view public-health services as important and part of their role, various organizational barriers limit their involvement [21]. These results confirm the profession's widespread acceptance of community pharmacists' changing role from traditional dispensing duties to greater involvement in health promotion and prevention and its understanding of the importance of providing these services.

Evidently, pharmacists and the population at large would welcome greater involvement of community pharmacies in health-promotion and preventive services [21,22]. Our results suggest that the development of future public-health programs in community pharmacy should focus on the continuity of care, maximizing the expertise of other health care professionals who may be present in the pharmacy, and overcoming organizational barriers. Such programs need to be well integrated within the primary-care system through effective communication and collaboration with other health care providers, and they should be supported by clinical tools, such as collective prescriptions, to optimize the contribution of pharmacists.

In a study evaluating the impact of a community pharmacy-based smoking cessation program in Northern Ireland, the involvement of pharmacies was especially low: only 19% of recruited pharmacies enrolled the required number of patients to participate in the study [7]. This is in line with the results of the present study, suggesting that although pharmacists may envision an ideal level of involvement in health-promotion and preventive services, this vision is often not translated into their actual practice. In formal clinical trials where the level of involvement of pharmacists in specific public-health activities may be considered as ideal, beneficial impacts have been identified, namely in the area of smoking cessation [6,7], hypertension [9,10], dyslipidemia [9], diabetes [8,9], and sexual health [11]. Integrating primary-care pharmacists and pharmacies into public-health programs should be considered a valuable option for optimizing population health. It is therefore crucial to better understand the barriers and facilitators of greater involvement of pharmacists in public health activities.

Similarly again to our results, studies have identified key barriers to the involvement of community pharmacists in health promotion and prevention. They include the lack of time, insufficient human resources, difficult access to patients' physicians, lack of skills and/or instrumentation, lack of compensation for prevention acts, and lack of space [1,23-25]. Other factors limiting the provision of such activities in current practice that were found in the literature but did not stand out in our study include the general public's lack of awareness of pharmacy's role in health promotion and prevention, lack of access to the full patient record, confidentiality concerns, and defensive or uncooperative patients [23-26]. It is therefore to be expected that overcoming these barriers will require reorganizing not only community-pharmacy practice but also the health care system in order to better integrate pharmacists into the provision of preventive services. This reorganization will doubtless require agreement, commitment and engagement by all pharmacy stakeholders in addition to financial investments.

Two studies have identified facilitators of practice change in community pharmacy [27,28]. These factors included government policy; remuneration for service delivery; communication and teamwork; leadership; task delegation; external support or assistance; reorganization of the structure and function of the pharmacy; professional satisfaction or competitiveness; communication and collaboration with physicians; and patient expectations regarding the services to be offered. Future public-health programs in community pharmacy will also need to consider these factors in order to facilitate practice change.

This study has some limitations. No data were collected regarding non-respondents; it is therefore possible that the pharmacists who returned the completed questionnaire are more motivated or interested than the non-respondents are and that involvement in health promotion and prevention may thus have been overestimated. A social-desirability bias may also have contributed to overestimate the ideal and actual prevention practices. Finally, the length of the questionnaire may have contributed to reduce the response rate. Nonetheless, despite random sampling and the absence of any telephone contact, the response rate was relatively high. Moreover, comparison with statistics compiled by the OPQ on gender and pharmacist status showed that our sample was representative of community pharmacists working in Quebec.

## Conclusions

In conclusion, community pharmacists strongly believe they should play a significant role in health promotion and prevention. However, given the many organizational barriers limiting their current public health activities, a wide gap exists between their ideal and actual levels of involvement.

## Methods

### Study design

In this cross-sectional study, a self-administered questionnaire was mailed to a random sample of 1,250 community pharmacists practicing in Montreal and surrounding areas from December 8, 2010, to February 23, 2011. The study was approved by the Research Ethics Committee of the *Centre de santé et de services sociaux de Laval *(Quebec, Canada). As an incentive, each respondent was eligible for a random draw of one of 10 prizes of $500.

### Sampling procedures

Using the OPQ's 2010 listing, which includes work-place or home addresses, 1,887 community pharmacists were identified in Montreal, Laval, Laurentides, Lanaudière, Montérégie, Estrie, and Outaouais. Pharmacists working in acute- and chronic-care hospitals or institutions are not included in the list. A random sample of 1,250 pharmacists weighted by the number of pharmacists in each region was constituted: Montreal (n = 481 or 38.5%),

Laval (n = 119, 9.5%), Laurentides (n = 125, 10.0%), Lanaudière (n = 103, 8.2%), Montérégie (n = 313, 25.0%), Estrie (n = 64, 5.1%), and Outaouais (n = 45, 3.6%).

### Mailings

A modified version of Dillman's tailored design method [29] was used to send the questionnaire to community pharmacists. The procedure comprised: 1) a personalized letter of invitation describing the study; 2) a first mailing of the questionnaire one week after the invitation; 3) a reminder postcard sent to non-respondents two weeks after the first questionnaire mailing; 4) a second mailing of the questionnaire to non-respondents two weeks after the postcard; and 5) a third mailing of the questionnaire to non-respondents three weeks after the second questionnaire mailing. Questionnaire mailings included a prepaid, preaddressed return envelope.

### Questionnaire

The questionnaire was written in French and is available online [30]. The questionnaire was not translated in English because in Quebec, all pharmacy programs including bridging programs for pharmacists from outside of Quebec are offered in French; all pharmacists in Quebec must therefore read, write and speak French. It consisted of 28 questions (in 11 pages plus a cover letter) and took around 25 minutes to complete. These questions were inspired by, but not restricted to, two previous questionnaires regarding the role of community pharmacists in health education and disease prevention [1,26], and questions were reviewed for relevance by all authors. The questionnaire was pretested for comprehension, language, relevance, and acceptability with a convenience sample of five volunteer community pharmacists who were not considered for participation in the study.

The first set of questions documented community pharmacists' ideal level of involvement in providing health-promotion and preventive services. Pharmacists were then asked to indicate which services were actually offered in their pharmacy, the employees involved, and the frequency and duration of the services. Finally, we documented their perceptions of the barriers to providing health-promotion and preventive services in community pharmacy and their opinion regarding the most appropriate health professionals to provide them. We also documented the characteristics of pharmacists, pharmacies and their clientele.

Pharmacists working in more than one community pharmacy were asked to refer to the one in which they worked most of the time. Respondents' characteristics were compared with the characteristics reported for Quebec pharmacists in the OPQ's annual report [31].

### Statistical analyses

The characteristics of pharmacists and their community pharmacies were described using means (and standard deviations) for continuous variables and proportions for discrete variables. For the questions on community pharmacists' role in health promotion and prevention and on the barriers to the provision of such services, the proportions of respondents selecting each possible answer were computed. With a sample of 571 respondents, the margin of error is estimated to be ± 4.1% 19 times out of 20, assuming a probability of 50%. Analyses were performed using SPSS software, version 19.0 (SPSS Inc., Chicago, IL).

## Abbreviations

CLSC: Local community service centre; OPQ: Ordre des pharmaciens du Québec

## Competing interests

MCL, SP and ND declare no competing interests. LL has unrestricted educational and research funding from Amgen Canada, AstraZeneca Canada Inc., Janssen-Ortho Inc., Merck Frosst Canada Ltd., Pfizer Canada Inc., Purdue Pharma Canada, and Leo Pharma.

## Authors' contributions

MCL participated in the design of the study, performed the data collection, carried out the statistical analyses, interpreted the data and wrote the article as first author. SP, ND and LL all contributed to the design of the study, interpreted the data, revised critically the manuscript and approved the final version.

## Financial disclosure

Réseau québécois de recherche sur l'usage des médicaments (RQRUM) and Agence de la santé et des services sociaux de Laval

## Pre-publication history

The pre-publication history for this paper can be accessed here:

http://www.biomedcentral.com/1471-2458/12/192/prepub
